# Effects of Concentrations on the Transdermal Permeation Enhancing Mechanisms of Borneol: A Coarse-Grained Molecular Dynamics Simulation on Mixed-Bilayer Membranes

**DOI:** 10.3390/ijms17081349

**Published:** 2016-08-18

**Authors:** Xingxing Dai, Qianqian Yin, Guang Wan, Ran Wang, Xinyuan Shi, Yanjiang Qiao

**Affiliations:** 1 School of Traditional Chinese Medicine, Beijing University of Chinese Medicine, No. 6 of Zhonghuan South Road, Wangjing, Chaoyang District, Beijing 100102, China; jolly_dai@126.com (X.D.); zmnh08830@126.com (Q.Y.); 2 Key Laboratory of TCM-information Engineer of State Administration of TCM, No. 6 of Zhonghuan South Road, Wangjing, Chaoyang District, Beijing 100102, China; 3 Beijing Key Laboratory of Manufacturing Process Control and Quality Evaluation of Chinese Medicine, No. 6 of Zhonghuan South Road, Wangjing, Chaoyang District, Beijing 100102, China; 4 School of Traditional Chinese Medicine, Capital Medical University, No. 10 of Xitoutiao Outside Youanmen, Fengtai District, Beijing 100069, China; wungung91@163.com (G.W.); wangran2017@sina.com (R.W.)

**Keywords:** molecular dynamics simulation, coarse-grained force field, transdermal drug delivery system, permeation enhancer

## Abstract

Borneol is a natural permeation enhancer that is effective in drugs used in traditional clinical practices as well as in modern scientific research. However, its molecular mechanism is not fully understood. In this study, a mixed coarse-grained model of stratum corneum (SC) lipid bilayer comprised of Ceramide-*N*-sphingosine (CER NS) 24:0, cholesterol (CHOL) and free fatty acids (FFA) 24:0 (2:2:1) was used to examine the permeation enhancing mechanism of borneol on the model drug osthole. We found two different mechanisms that were dependent on concentrations levels of borneol. At low concentrations, the lipid system maintained a bilayer structure. The addition of borneol made the lipid bilayer loosen and improved drug permeation. The “pull” effect of borneol also improved drug permeation. However, for a strongly hydrophobic drug like osthole, the permeation enhancement of borneol was limited. When most borneol molecules permeated into bilayers and were located at the hydrophobic tail region, the spatial competition effect inhibited drug molecules from permeating deeper into the bilayer. At high concentrations, borneol led to the formation of water pores and long-lived reversed micelles. This improved the permeation of osthole and possibly other hydrophobic or hydrophilic drugs through the SC. Our simulation results were supported by Franz diffusion tests and transmission electron microscope (TEM) experiments.

## 1. Introduction

Transdermal drug delivery systems (TDDS) are known for their advantages of bypassing first-pass liver metabolism, gastrointestinal irritation, improved higher bioavailability, better patient compliance and reduced side effects [1,2]. A common problem with TDDS design is the transport barrier of skin stratum corneum (SC). To solve this problem, numerous techniques have been used, such as the use of permeation enhancers (PEs) [3,4,5,6]. Essential oils (or volatile oils) are a type of natural permeation enhancers that can effectively promote the permeation of both hydrophilic and hydrophobic drugs [7,8,9]. Essential oils are biocompatible with commonly-used chemical synthetics PEs because they are safe, non-toxic, pharmacologically inert, non-irritating, hypo-allergenic and have a wide range of pharmacological functions, such as anti-inflammatory and anticancer applications [10,11,12,13].

Natural borneol (BO) is a monoterpenoid component extracted from the essential oil of *Cinnamomum camphora* (L.) Presl. Prior pharmacological studies showed that borneol has anti-inflammatory, antinociceptive and antibacterial uses as well as other biological activities [14,15,16]. According to Traditional Chinese Medicinal (TCM) theory, borneol is not only used as a drug, but also as an excipient for other drugs (referred to as “YAO FU HE YI” in TCM theory). Permeation enhancement is an important mechanism for the excipient effect of borneol. Many drugs, such as berberine, geniposide, ribavirin, tobramycin, ligustrazine, and loratadine [17,18,19,20,21], have significant improvements in permeation when used with borneol. However, most prior studies focused on the permeation enhancing behavior of borneol in in vitro tests, but did not examine the involved molecular mechanisms.

Interactions with lipid bilayers are the most important mechanisms for the permeation enhancing effects of borneol [22]. To fully understand these mechanisms, our team used the coarse-grained molecular dynamic (CG-MD) simulation method to study the interactions of borneol on dipalmitoyl phosphatidylcholine (DPPC) phospholipid membranes, since borneol can enhance drug permeation not only through SC but also through mucosa and other bio-membranes. The main idea of CG-MD is to coarse-grain the familiar atomistic representation of the molecule to gain orders of magnitude in both length and time scale relative to traditional atomistic scale simulation [23]. The simulation is based on Newton’s equation of motion and is commonly used to study biological systems [24,25,26,27,28]. It also provides insight into the thermodynamics and dynamics properties of mesoscale substances (1–1000 nm). Similar to atomistic simulations, there are interactions between coarse-grained particles, and these interactions are often called coarse-grained force fields. The Martini force field, developed by Marrink and coworkers in 2007, is a special coarse-grained force field [29]. Because of its portability and expansibility, Martini force field has been widely used in many studies of biomolecules, such as lipids, polymers, proteins, carbohydrates, and so on [29,30,31,32,33,34].

In our previous study, the CG-MD simulation was useful to show the influence of borneol on DPPC membranes and explained the permeation enhancing mechanisms of borneol. However, the lipids in SC are different from those in DPPC membranes. SC lipids are comprised of ceramides (CER), cholesterol (CHOL) and free fatty acids (FFA), and organized in lamellar layers around the corneocytes [35]. This structure is important for SC lipid function. Ceramides are the major components of SC. Over 300 ceramides have been identified in SC with fatty acid lengths varying from 16 to 34 carbons. Among them, Ceramide-*N*-sphingosine (CER NS) is the most abundant species in human SC, and the most commonly studied ceramide in lipid model of SC [35,36,37]. Cholesterol is also an essential component of the SC. Its presence reduces the ordering of ceramide tails and simultaneously increases lipid fluidity. As a result, the range of phase transition temperatures (Tm) is enlarged. Free fatty acids, however, can increase the density and compact the ordering of the hydrophobic lipid tails [38,39]. In order to get more accurate results, we used a mixed CG model of SC lipid bilayer comprised of CER NS 24:0, CHOL and FFA 24:0 (2:2:1) [40] (the properties of this molar proportion lipid layer have been validated by Das and coworkers in 2009 [41,42]) to measure the enhanced transdermal permeability effects. We conducted several CG-MD simulations based on Martini force field to investigate the interaction of borneol with the SC lipid model we built before [40].

Osthole (OST), an antibacterial drug, is often used with borneol in traditional Chinese transdermal preparations to treat surgical diseases such as gynecologic inflammation, tinea pedis, and psoriasis [43]. It is known that the permeability of osthole can be enhanced when used with borneol [44]. In this work, osthole was used as the model drug to study the enhanced transdermal permeation effects and mechanism of borneol by both theoretical and experimental methods. The simulation results were verified by Franz diffusion tests and transmission electron microscope (TEM) experiments.

## 2. Results and Discussion

### 2.1. Interaction of Borneol with SC Lipid Bilayer

**Figure 1:**
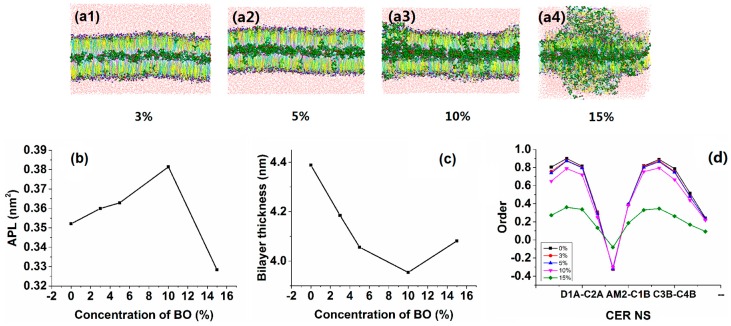
Morphologies and corresponding analyses of lipid bilayer systems at different borneol concentrations by CG-MD simulations: (**a**) morphologies of the lipid bilayer at (1) 3%, (2) 5%, (3) 10% and (4) 15% of borneol; (**b**) APL analysis; (**c**) bilayer thickness analysis; and (**d**) order parameter analysis for CER NS.

Figure 1(a1–a4) shows the CG simulation results of bilayer with different concentrations of borneol. We found that the lipid bilayer showed different morphological features at different concentrations of borneol, which indicated the different permeation enhancing mechanisms. Below the concentration of 10%, the lipid systems maintained a whole bilayer structure with most of the borneol molecules located at the hydrophobic lipid tail area (Figure 1(a1–a3)). The calculation of average area per lipid (APL), bilayer thickness and order parameters of CER NS (Figure 1b–d) showed that with the increasing of borneol concentration, there was a correlation between increased APL and decreased bilayer thickness and order. The borneol molecules permeated into lipid bilayer and occupied the intermolecular or intramolecular space of lipid molecules, which resulted in the increased APL. As such, borneol disturbed the orderly arrangement of lipid tails and made them easier to bend, which caused the bilayer thickness to decrease (Figures S1–S3 in Supplementary Materials). This much loosened structure increased the permeation of agents through the bilayer. By using fluorescence recovery after photo-bleaching technology (FRAP), Fu [45] demonstrated that borneol improved the fluidity of lipid membranes and that the fluidity increased as the borneol concentration increased. Fu’s finding is consistent with our simulation results.

**Figure 2:**
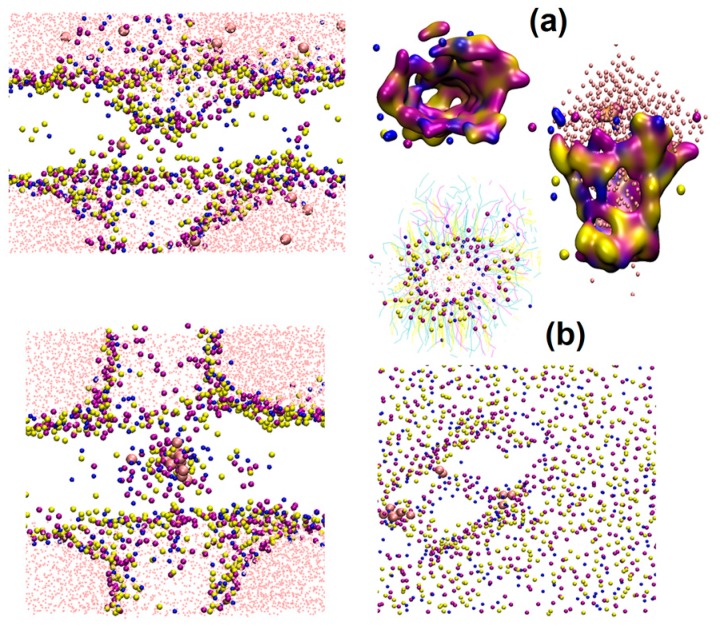
Detailed structures of water pores and reversed micelles formed by borneol in lipid bilayers by CG-MD simulations: (**a**) water pore; and (**b**) reversed micelle.

At high concentrations (above 15%), the bilayer structure was dramatically altered (Figure 1(a4)). The order of lipids dropped significantly (Figure 1d). The borneol molecules extracted the lipids from the bilayer and formed water pores and reversed micelles. Their detailed structures are shown in Figure 2. By using attenuated total reflectance-Fourier transform infrared spectroscopy (ATR-FTIR) to monitor the borneol-induced alteration in molecular organization of SC lipids, Yi Qifeng found borneol could perturb the structure of SC lipid alkyl chains, and extract part of SC lipids, resulting in the alteration in the skin permeability barrier [22]. These results are consistent with our findings. It has been reported that water pores and reversed micelles can contribute to the permeation enhancing effect of drugs, especially hydrophilic agents and ions [46]. As for hydrophobic agents like osthole, the effect of these structures will be discussed later. 

**Figure 3:**
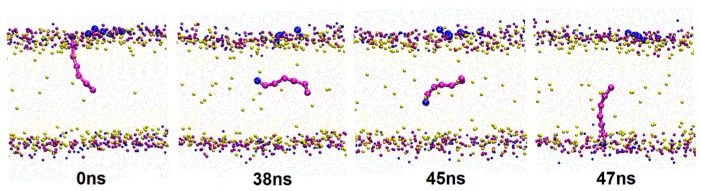
A typical lipid flip-flop process by CG-MD simulations. To show the dynamic process clearly, water and lipid tail beads were hidden in the figures.

The molecular trajectory show that along with the permeation of borneol, some lipid molecules in bilayer spontaneously experienced transmembrane flip from one leaflet to another (flip-flop). A typical flip-flop process of a free fatty acid molecule is shown in Figure 3. Lipid flip-flop is a common and important phenomenon involved in many cellular properties and functions, such as membrane mechanical stability [47,48]. Prior studies found that one of the important mechanisms of lipid flip-flop in protein-free membranes was associated with the water pore. Environmental disturbances on the membrane, such as transmembrane ion concentration gradients, transmembrane tension changes and nanoparticle diffusion can initiate the formation of a water pore. In addition to lipids, cholesterol also experiences transmembrane flip in the membrane. MD studies by Bennett [47] revealed that stronger affinities between cholesterol and surrounding lipids lead to higher energy for cholesterol to transmembrane flip. There may be two mechanisms for the lipid flip-flop caused by the addition of borneol. First, the permeation of borneol molecules destroyed the hydrogen bond network of the bilayer system, which weakens the interaction between free fatty acids and other constituent molecules and allows the lipid molecules to easily flip; Second, the water pore caused by borneol mediated the flip. The flip-flop may reduce the mechanical strength of the bilayer membrane that further improves the permeability of drugs.

### 2.2. Permeation Enhancing Effect of BO on OST

Osthole is the active ingredient derived from *Cnidium monnieri* (L.) Cuss and has a broad-spectrum antimicrobial effect. It is often used in traditional Chinese topical preparations to treat bacterial diseases. Prior studies reported that borneol improved the permeation of osthole through skin. The enhanced effect is better than the commonly used chemical synthesis PE azone [44,49]. In our study, we simulated the permeation behavior of osthole with and without borneol in lipid bilayer to study the permeation enhancing effect of borneol on osthole. The concentration of osthole was fixed at 10%.

#### 2.2.1. CG-MD Simulation Studies

**Figure 4:**
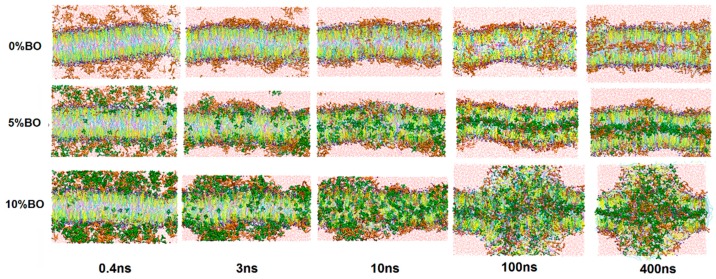
Morphology evolution of lipid bilayer systems with time at different concentrations of borneol by CG-MD simulations. The concentration of osthole was fixed at 10%.

Figure 4 shows the morphology evolution of lipid bilayer systems with time. The osthole concentration was fixed at 10% to study the borneol concentration effect on the permeation behavior of osthole.

**Figure 5:**
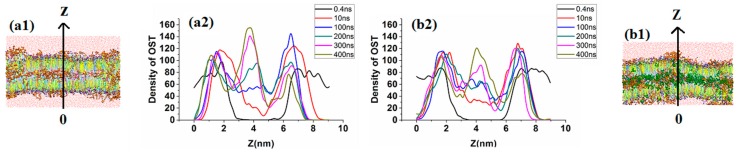
(1) Lateral views and (2) corresponding density profiles of osthole in *z*-axis direction in bilayer systems at different points in time by CG-MD simulations: (**a**) system without borneol; and (**b**) system with 5% borneol.

**Figure 6:**
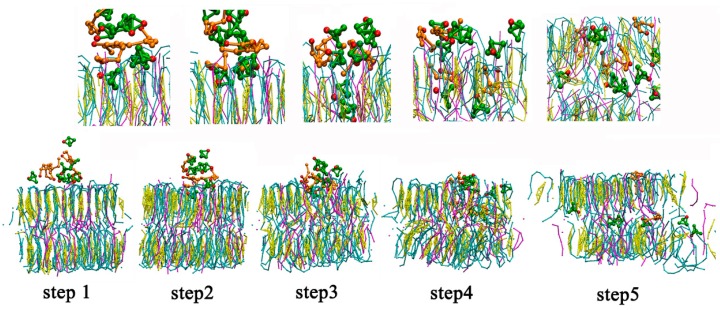
Permeation process of osthole molecules with “pull” effect of borneol during the first 100 ns of 5% borneol and 10% osthole system by CG-MD simulations.

When the concentration was under 10%, the lipids system still maintained a whole bilayer structure with a slight fluctuation. The comparison of systems with and without 5% borneol concentrations revealed that the borneol molecules had higher permeability than osthole because they have strong interaction with lipids, especially on the hydrophilic layer. After 10 ns of MD simulation, almost all of the borneol molecules permeated into the bilayer while most of the osthole molecules were still located at lipid–water interface (Figure 5). To evaluate the permeability of osthole with borneol, the density distribution of osthole along the vertical direction of bilayer (the *Z* direction) in systems is shown in Figure 6. The results indicated that, with and without the borneol, osthole could gradually diffuse into the lipid layer over time. During the first 100 ns, the osthole density in the borneol containing system was higher, which indicated faster diffusion. However, it was also higher in the borneol excluding systems after 100 ns. Therefore, the borneol could inhibit the permeation of osthole after 100 ns. However, no data exist regarding the inhibition effects of borneol. To explain this phenomenon, we studied the whole dynamic trajectory (Figure 6 and Figure 8). Osthole had strong hydrophobicity (logP = 3.8). Therefore, the hydrophilic head layers were the main barrier for the permeation of osthole. During the first 100 ns, borneol interacted with lipid layers preferentially because of its stronger interaction with lipid molecules. They acted on the polar head groups of lipids by their polar oxygen groups and destroyed the hydrogen bond network of head groups and weakened the bilayer. Then, the borneol molecules pulled the osthole molecules and permeated into the tail region through hydrophobic interactions, as illustrated in Figure 8. That may be the reason for the faster diffusion of osthole during the first 100 ns. This “pull” effect was previously reported as a possible permeation enhancing mechanism for 1,8-cineole [50], which is another natural terpenoid PE similar to borneol. When the system reached equilibrium (after 100 ns), the ordering of lipids somewhat recovered. This indicated that the lipid disturbance caused by borneol at low concentrations was reversible. Nearly all of the borneol molecules permeated into the bilayer and were located at the hydrophobic tail region. The “pull” effect disappeared. However, the hydrophobic tail region was also the main region for osthole to locate. The occupation of borneol made osthole difficult to permeate into bilayer further, and therefore it caused the inhibition effect. Since the inhibition effect was not previously studied, some in vitro permeation tests were conducted to verify the simulation results. 

**Figure 7:**
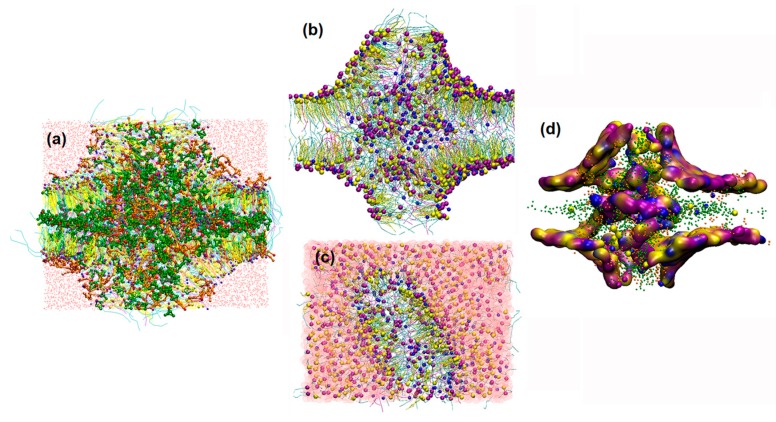
Detail illustration of osthole molecules interacting with water pores and reversed micelles formed by 10% borneol, 10% osthole and SC lipids by CG-MD simulations: (**a**) lateral view of the bilayer with all the constituent molecules; (**b**) lateral and (**c**) vertical views of the bilayer with lipid and solvent molecules; and (**d**) lateral view of the bilayer with hydrophilic head groups shown in density map and hydrophobic alkyl chains hidden.

When the concentration of borneol was above 10%, the lipid bilayer structures were dramatically perturbed. Water pores and reversed micelles were found formed at this concentration, similar to the structures mentioned in Section 3.1. As shown in Figure 7, borneol extracted part of SC lipids and induced some lipids to drill into the bilayer, forming reversed micelles. This facilitated the contact of osthole molecules with the hydrophobic alkyl chains of lipids. The disordered condition of the hydrophobic region was further conducive to the diffusion of osthole. Besides, osthole molecules were found that they could also permeate through the water pores. These mechanisms jointly resulted in the permeation enhancement of osthole. It is worth noting that, the concentration (10%) of water pore and the reversed micelle in the system with osthole was lower than that (15%) in the system without osthole. This indicated that the addition of osthole could reduce the effective concentration of borneol. Therefore, when evaluating the dosage of PEs, the effects of drugs should be considered.

#### 2.2.2. In Vitro Permeation Studies

**Figure 8:**
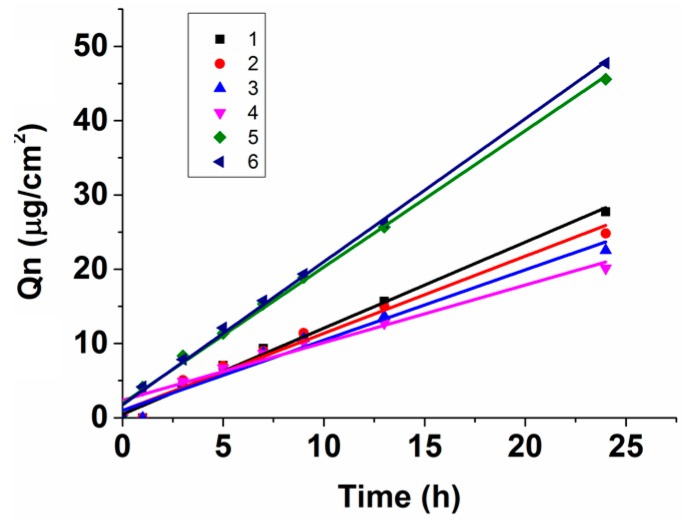
Cumulative amount of osthole in the presence of varying concentration of borneol and the corresponding zero-order kinetics equations.

The in vitro permeation data of osthole under different borneol concentrations obtained from Franz diffusion experiments is graphed as a function of cumulative amount *Q_n_* vs. time in Figure 10. The assessment parameters calculated from the *Q_n_*-*t* equations are shown in Table 1. We were pleased to find that borneol had two different effects on the permeation of osthole. At low concentrations (0.09%~0.54%), the *Q_n_* of osthole with borneol was lower than concentrations without borneol at all time points. The *PR* and *ER* also indicated that borneol inhibited the permeation of osthole through SC and that the inhibition rate increased as the concentration increased. The same decrease in the permeability coefficient *K_p_* in in vitro experiments and in the diffusion coefficient *D* in simulations was in accord with our previous calculations (Figure 8).

**Figure 9:**
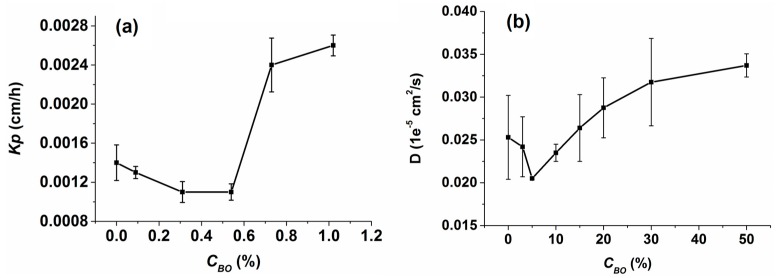
(**a**) Permeability coefficient *K_p_* of osthole obtained from in vitro experiments; and (**b**) diffusion coefficient *D* of osthole obtained from CG-MD simulations. Either *K_p_* or *D* reflects the diffusion rate of osthole in the corresponding lipid bilayer systems.

When the concentration was above 0.54%, the *Q_n_* of osthole sharply increased. The *PR* and *ER* value indicated that borneol significantly enhanced the permeation of osthole through SC. We searched relevant literature and found that most reported effective enhancing concentrations of borneol were above 0.5% [44,51,52,53]. This may just be the results of the bilayer morphology changing, as illustrated in the simulations. Severe lipid disturbance coupled with the formation of water pores and reverse micelles destroyed the reservoir function of SC and caused it to weaken so that the osthole could easily permeate. A significant increase the in diffusion rate of osthole at high concentrations was observed in both the in vitro experiments and in the simulations (Figure 9). The inflection point indicted the bilayer morphology changing concentration. The concentrations in the in vitro experiments did not correspond with the concentrations in the simulations because we ignored non-essential details in the CG-MG simulations so that relevant details could be determined while maintaining computer efficiency. Thus, the concentrations in simulations were slightly higher than the ones in vitro, but the variation tendency was the same.

#### 2.2.3. TEM Studies

**Figure 10:**
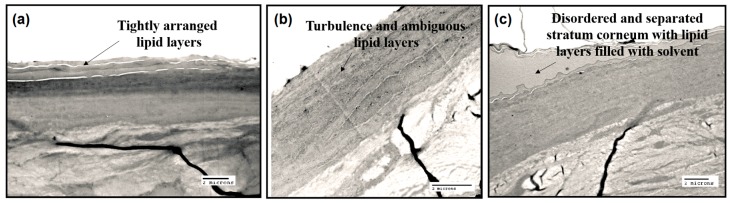
Transmission electron microscope (TEM) views of hairless rat skins with 10% osthole and different concentrations of borneol treated at 35 °C for 24 h: (**a**) 0%; (**b**) 0.54%; and (**c**) 1.02%. Bar = 2 μm.

TEM studies were performed to further assess the effects of borneol on the permeation of osthole. As shown in Figure 10a, in the absence of borneol, the SC exhibited the tightly arranged corneocytes with a surrounding packed lamellar lipid layers. After treatment with 0.54% borneol for 24 h, the SC became irregular. The turbulence and ambiguous lipid layers images are shown in Figure 10b. Focal dilutions occurred within the intercellular space, since the permeation of borneol into hydrophobic tail region disordered the arrangement of lipid molecules. However, when the concentrations increased to 1.02%, the lipid layers of stratum corneum were completely disordered and separated (Figure 10c). That may be the results of the water pores formed by borneol stimulated the permeation of solution containing osthole. In conclusion, the TEM studies verified our theory that intercellular lamellar bilayer turbulence and water pore formation induced by borneol enhanced the permeability of osthole.

## 3. Materials and Methods

### 3.1. Simulation Method

**Figure 11:**
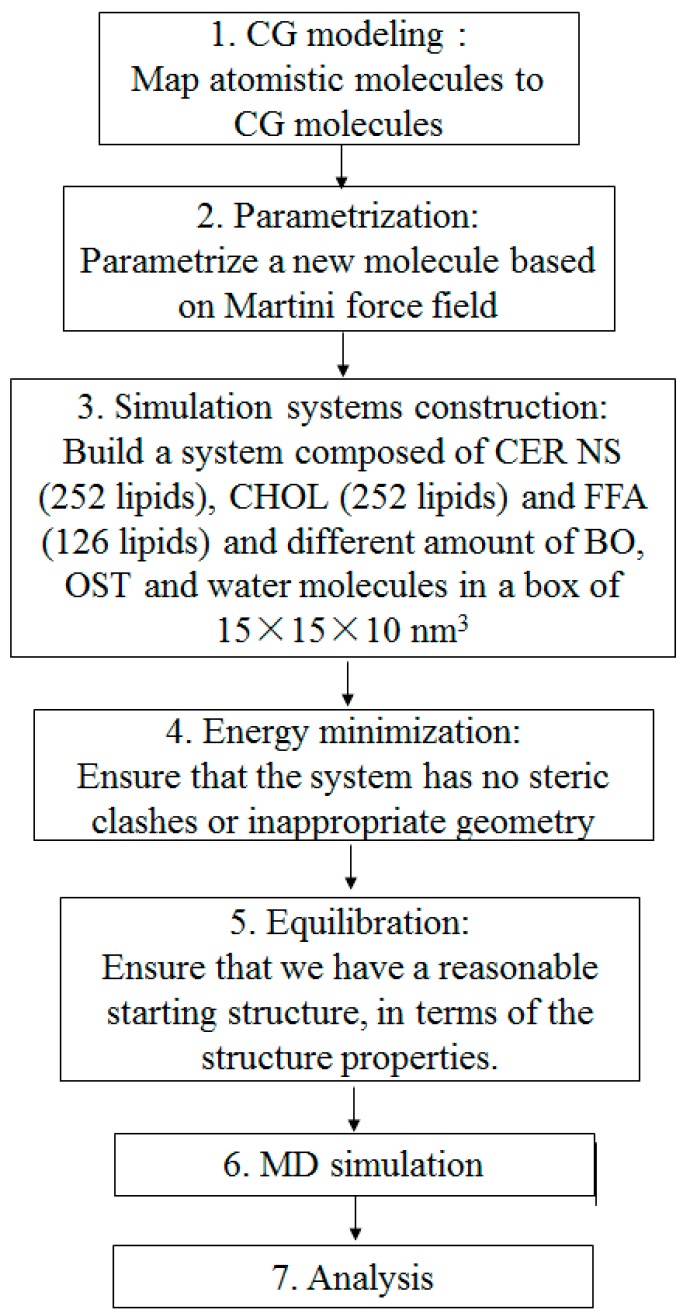
The flow chat of the coarse-grained molecular dynamic (CG-MD) simulation process.

Figure 11 shows a typical CG-MD simulation process in this study. All simulation systems were built using the Packmol package [54] and the figures depicting molecules were generated by Visual Molecular Dynamics (VMD) [55]. The simulations were conducted using the GROMACS software package (version 4.6.3) [56].

#### 3.1.1. CG Models

**Figure 12:**
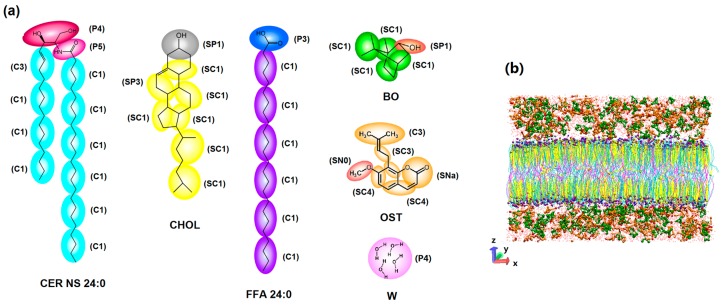
CG models for simulation systems: (**a**) the chemical structure and CG mapping for Ceramide-*N*-sphingosine (CER NS), cholesterol (CHOL), free fatty acids (FFA), borneol (BO), osthole (OST) and solvent water W based on Martini force field; and (**b**) a side view of a typical simulation system (membrane with 5% BO and 10% OST in water).

The CG models and their force field parameters used in this simulation were based on the Martini force field [57]. The Martini model is based on a four-to-one mapping (on average four heavy atoms are represented by a single interaction center), and a two/three-to-one mapping method is used for ring structures. Our simulation systems involved six molecules: CER NS (24:0), CHOL, FFA (24:0), BO, OST and solvent water (W). Their chemical structures and CG mappings are shown in Figure 12a and their interaction parameters are shown in Table S1 in the Supplementary Materials. Here, the CG models of borneol and osthole are new models developed by our team. The validation of these CG models is also shown in Section S2 in the Supplementary Materials.

#### 3.1.2. Initial Bilayer Structures

An initial mixed-bilayer membrane composed of CER NS (252 lipids), CHOL (252 lipids) and FFA (126 lipids) in a molar ratio of 2:2:1 was built in a box of 15 × 15 × 10 nm^3^. Coarse-grained water molecules (W) were filled into the box. The system energy was minimized using the method of steepest descent to remove the bad contacts between molecules. The minimized structure was equilibrated for 100 ns at 310 K. The structural properties of this mixed-bilayer system are shown in Section S3 of the Supplementary Materials. This equilibrated structure was further used as a starting structure to study the permeation behavior of OST at different concentrations of BO. The bilayer systems with different concentrations of BO and OST in water were also built using the Packmol package (Figure 12b).

#### 3.1.3. Simulation Details

All simulations were conducted in the NVT ensemble. The simulation temperature was set at 310 K by using the Berendsen temperature coupling with a time constant of 1.0 ps. The pressure was controlled by the Berendsen barostat and semi-isotropic pressure coupling with a constant time of 3.0 ps and compressibility of 4.5 × 10^−4^/bar. The method for both electrostatics and Van der Waals had the cut-off length of 1.2 nm. The time step was 20 fs and the total simulation time was 400 ns, which was sufficient for the simulation systems to reach equilibrium (see Section S4 in Supplementary Materials).

### 3.2. Verification Experiments

#### 3.2.1. Materials

Borneol and osthole (purity > 98%) were purchased from National Institutes for Food and Drug Control (Beijing, China) and 2.5% of glutaraldehyde was provided by Biotopped Life Science (Beijing, China). Methanol and acetonitrile of HPLC grade were supplied by Thermo Fisher Scientific (Beijing China). All other reagents were readily available from various commercial sources at analytical grade.

#### 3.2.2. In Vitro Permeation Studies

##### Preparation of Osthole Solutions with Different Concentrations of Borneol

Because osthole has poor aqueous solubility, so 80% propanediol, which does not influence SC [55,56,57,58], was used to dissolve the osthole in the in vitro experiments. The solution was placed in an ultrasonic cleaner for 15 min followed by another equilibrium process for 24 h at 35 °C. The solution was filtered through a 0.45 μm millipore filter, and the final concentration of osthole was 0.1%. The osthole solution was then used as solvent to prepare different concentrations of borneol solutions. The concentrations of borneol were 0.09%, 0.31%, 0.54%, 0.73% and 1.02%.

##### Preparation of Skin

SPF male Sprague-Dawley rats weighing 190–210 g were purchased from Sibeifu Laboratory Animals Co., Ltd. (Beijing, China). The rats were euthanized after excessive ethyl ether anesthesia. The abdominal skin was excised after the hair was carefully trimmed. The subcutaneous fat and connective tissue were removed. The skin samples were washed with ultrapure water and a 0.9% sodium chloride solution and then equilibrated at 35 °C for 1 h in a receptor medium (80% propanediol) in Franz diffusion cells. All experiments on the animals were conducted in conformity with institutional guidelines for the care and use of laboratory animals in Beijing University of Chinese Medicine, Beijing, China.

##### Skin Permeation

Franz diffusion cells with an effective diffusion area of 0.785 cm^2^ and a receptor volume of 10 mL were used to perform the in vitro skin permeation studies. One-milliliter osthole solutions with different concentrations of borneol were added to corresponding donor chambers. The receptor chambers were filled with 80% propanediol as receptor medium which was maintained at 35 ± 0.5 °C with a magnetic stirrer at 300 rpm. A 1 mL receptor medium was sampled at predetermined time intervals (0, 2, 4, 6, 8, 10, 12, and 24 h) and then the same volume of pure medium was immediately added to the receptor chamber. All solution samples were filtered through a 0.45 μm millipore filter and stored at 4 °C for HPLC later analysis.

##### HPLC Analysis

The quantitative determination of osthole was measured with an HPLC system (Agilent 1100, Agilent, Inc., Santa Clara, CA, USA) using acetonitrile–water (65:35 *v*/*v*) as mobile phase at a flow rate of 1.0 mL/min. The injection volume was 10 μL. A Waters Xbridge C18 column (250 mm × 4.6 mm, 5 μm, Waters, Inc., Hong Kong, China) was used. The UV detector wavelength was set at 322 nm and the column temperature was maintained at 35 °C.

##### Important Assessment Parameters

The main parameters used in this paper to assess the permeation enhancing effect of borneol were: the cumulative amount *Q_n_* (μg/cm^2^), the permeability constant *J* (μg/cm^2^·h), the permeability coefficient *K_p_* (cm/h), the enhancement ratio *ER* and the permeation ratio *PR*.

The quantity of drugs that permeated through SC is presented as cumulative amount *Q_n_* (μg/cm^2^) and is calculated using the following formula:

(1) $$Q_{n}=\frac{\left(C_{n}\;\times \;V_{r}+\;\sum_{i=1}^{n-1}C_{i}\;\times \;V_{i}\right)}{A}$$

where *C_n_* is the drug concentration of the receptor medium at each sampling time, *C_i_* is the drug concentration at *i*th sampling point, *V_r_* and *V_i_* were the volumes of receptor solutions and samplings, respectively, and *A* was the effective diffusion area of skin.

The zero-order permeating kinetics equation (*Q*-*t*) is obtained by regressing the cumulative amount on time:

(2) Q=Jt+B

where the slope *J* (μg/cm^2^·h) is the permeability constant.

The permeability coefficient of drugs is related to permeability coefficient *K_p_* (cm/h) using the following formula:

(3) $$K_{p}=J/C_{0}$$

where *C*_0_ (μg/ mL) is the initial concentration of drug.

The overall potency of PE is expressed as enhancement ratio (*ER*), a ratio of the *K_p_* value before and after enhancer treatment.

(4) $$ER=K_{pe}/K_{p}$$

where *K_pe_* is the *K_p_* value after treatment.

The enhancing effect of PE on drugs partitioning into the SC is described as permeation ratio (*PR*) and is calculated by:

(5) $$PR=\frac{Q_{n}\;\times \;A}{C_{0}\;\times \;V_{d}}\times 100\%$$

where *V_d_* is the donor volume.

##### Transmission Electron Microscope (TEM) Studies

Twenty-four hours after treatment, the skin samples were fixed in 2.5% glutaraldehyde. Before the TEM study, the samples were washed with phosphate buffer (pH 7.2) and fixed in 1% OsO_4_. The samples then were dehydrated in a graded series of acetone, embedded in a low-viscosity epon-epoxy mixture (provided by Beijing institute of traditional Chinese medicine) and sectioned. Thin sections were double stained with uranyl acetate and lead citrate and examined on a transmission electron microscope (JEOL JEM-1230, Tokyo, Japan) operated at an acceleration voltage of 80 kV.

## 4. Conclusions

Borneol is a natural permeation enhancer that is effective in drugs used in traditional clinical practices as well as in modern scientific research. However, its molecular mechanism is not fully understood. In this study, we discovered two different concentration dependent mechanisms of permeation that were enhanced by borneol using CG-MD simulations. At low concentrations, the lipid system maintained a bilayer structure. The addition of borneol made the lipid bilayer loosen enough for drug permeation. The “pull” effect of borneol also improved the permeation of drugs. However, for strongly hydrophobic drugs like osthole, permeation enhancement of borneol was limited. When borneol molecules permeated into the bilayer and were located at the hydrophobic tail region, the spatial competition effect inhibited drug molecules further into the bilayer. The results of Franz diffusion tests verified the permeation inhibition effect of borneol at low concentration (0%–0.54%). At high concentrations, borneol led to the formation of water pores and long-lived reversed micelles. This improved the permeation of osthole and possibly other hydrophobic or hydrophilic drugs through the SC. The results of Franz diffusion tests supported the permeation enhancing effect of borneol on osthole at high concentration (>0.054%). The TEM experiments further verified the lipid disturbance and pore-mediated pathway may be the permeation enhancing mechanism, as illustrated in our simulation experiments.

To date, borneol has been reported as a permeation enhancer for both hydrophobic and hydrophilic drugs, especially the former. Lipid disturbance and pore-mediated pathway were considered as the most important mechanisms as verified in our study. However, our findings were the first to recognize the “pull” effect mechanism as demonstrated in our simulations. Our study not only provided a detailed explanation of the molecular mechanisms on how the borneol enhanced the permeation of drugs like osthole, but also recommended that the effective concentration of borneol should be above 0.54%. In addition, the simulation method developed in this paper can be used in future studies of other percutaneous permeation systems.

## Figures and Tables

**Table 1:** Assessment parameters calculated from *Q_n_-t* equations.

No.	*C_BO_* (%)	*J_e_* (μg/cm^2^·h)	*K_p_* (cm/h)	*PR* (%)	*ER*
1	0.00	0.9571	0.0014	3.26	1.0000
2	0.09	0.8737	0.0013	2.94	0.9129
3	0.31	0.7464	0.0011	2.67	0.7798
4	0.54	0.7005	0.0011	2.38	0.7319
5	0.73	1.6125	0.0024	5.38	1.6848
6	1.02	1.7064	0.0026	5.64	1.7828

## Supplementary Materials

Supplementary materials can be found at www.mdpi.com/1422-0067/17/8/1349/s1.
