# Supplementary material for: Effects of Concentrations on the Transdermal Permeation Enhancing Mechanisms of Borneol: A Coarse-Grained Molecular Dynamics Simulation on Mixed-Bilayer Membranes
Source: Int J Mol Sci. 2016 Aug 18;17(8):1349. doi: 10.3390/ijms17081349 (PMC5000745; doi:10.3390/ijms17081349)
Supplement: Supplementary file 1 [file ijms-17-01349-s001.pdf]

Supplementary Materials: Effects of Concentrations on the Transdermal Permeation Enhancing Mechanisms of Borneol: A Coarse-Grained Molecular Dynamics Simulation on Mixed-bilayer Membranes

Xingxing Dai, Qianqian Yin, Guang Wan, Ran Wang, Xinyuan Shi and Yanjiang Qiao

1. More Simulation Results for the Interaction of Borneol with SC Lipid Bilayer

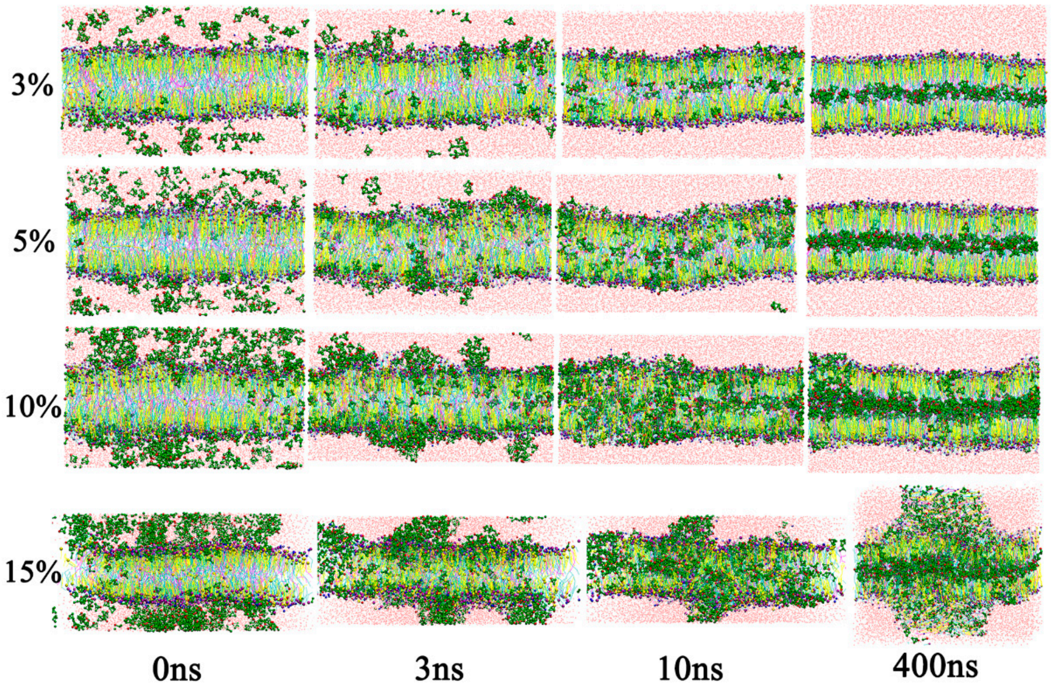

Figure S1. The morphology evolution of lipid bilayer systems with different concentrations of borneol added during the simulation time.

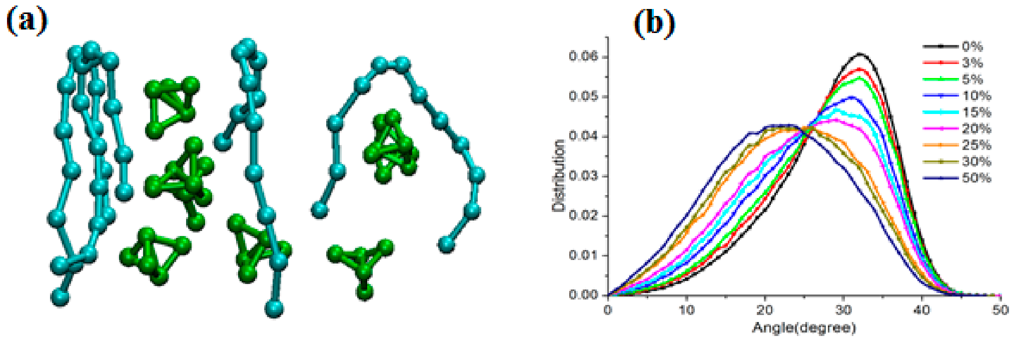

Figure S2. (**a**) Interaction configurations of borneol and ceramide molecules in lipid bilayers; (**b**) The angle distribution of ceramides at different concentration of borneol.

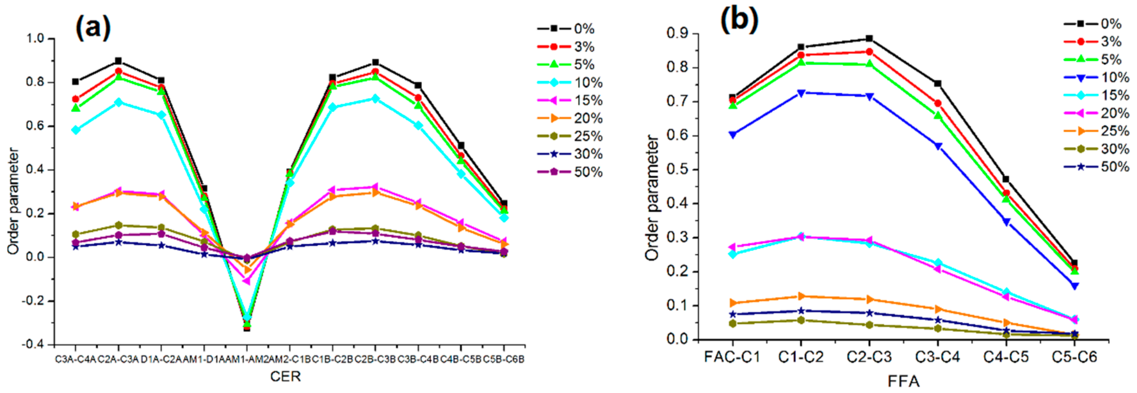

Figure S3. Order parameters of (**a**) ceramides and (**b**) free fatty acids at different concentrations of borneol.

2. CG Models

2.1. CG Mappings and Force Field Parameters

The main molecules involved in our simulation systems are ceramides NS 24:0 (CER NS), cholesterol (CHOL), free fatty acids 24:0 (FFA), borneol (BO) and osthole (OST). Their chemical structures and CG mappings are shown in Figure S4 and their force field parameters are shown in Table S1.

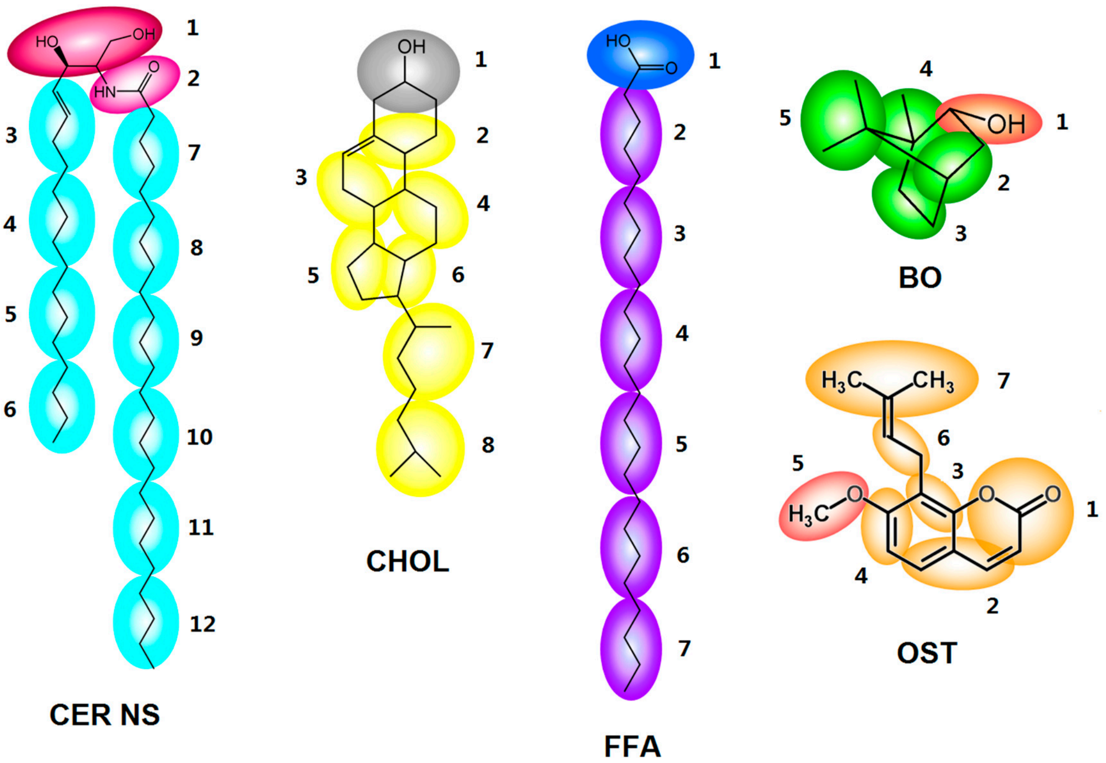

Figure S4. CG models of CER NS, CHOL, FFA, BO and OST.

Table S1. Force field parameters of CER NS, CHOL, FFA, BO and OST.

Molecules	Atoms	Name	Type	Bonds	R_{bond} (nm)	K_{bond} (kJ·mol^{-1}·nm^{-2})	Angles	θ_0 (deg)	K_{angle} (kJ·mol^{-1})	Dihedrals	φ_{pd} (deg)	$K_{dihedral}$ (kJ·mol^{-1}·rad^{-2})
CER NS	1	AM1	P4	1-2	0.27	20000	7-2-1	129	200			
	2	AM2	P5	1-3	0.29	20000	1-3-4	180	25			
	3	D1A	C3	3-4	0.47	1250	3-4-5	180	25			
	4	C2A	C1	4-5	0.47	1250	4-5-6	180	25			
	5	C3A	C1	5-6	0.47	1250	2-7-8	180	25			
	6	C4A	C1	2-7	0.37	20000	7-8-9	180	25			
	7	C1B	C1	7-8	0.47	1250	8-9-10	180	25			
	8	C2B	C1	8-9	0.47	1250	9-10-11	180	25			
	9	C3B	C1	9-10	0.47	1250	10-11-12	180	25			
	10	C4B	C1	10-11	0.47	1250						
	11	C5B	C1	11-12	0.47	1250						
	12	C6B	C1									
CHOL	1	ROH	SP1	1-2	0.242	20000	4-7-8	180	25	1-3-5-4	0	100
	2	R1	SC1	2-3	0.26	20000				1-3-5-7	0	100
	3	R2	SC3	2-4	0.341	20000				1-4-5-3	0	100
	4	R3	SC1	4-6	0.213	20000				3-5-7-4	0	100
	5	R4	SC1	4-7	0.544	20000				4-7-5-3	0	100
	6	R5	SC1	5-6	0.203	20000				2-1-3-4	-45	300
	7	C1	SC1	6-7	0.368	20000				2-4-3-1	45	300
	8	C2	C1	7-8	0.425	1250				6-4-5-7	-45	300
				1-3	0.493	constraint				6-7-5-4	45	300
				1-4	0.604	constraint						
				3-4	0.272	constraint						
				3-5	0.346	constraint						
				4-5	0.294	constraint						
				5-7	0.406	constraint						

Table S1. *Cont.*

Molecules	Atoms	Name	Type	Bonds	R_{bond} (nm)	K_{bond} (kJ·mol⁻¹·nm⁻²)	Angles	θ_0 (deg)	K_{angle} (kJ·mol⁻¹)	Dihedrals	φ_{pd} (deg)	$K_{dihedral}$ (kJ·mol⁻¹·rad⁻²)
FFA	1	FAC	P3	1-2	0.37	20000	1-2-3	180	25			
	2	C1	C1	2-3	0.47	1250	2-3-4	180	25			
	3	C2	C1	3-4	0.47	1250	3-4-5	180	25			
	4	C3	C1	4-5	0.47	1250	4-5-6	180	25			
	5	C4	C1	5-6	0.47	1250	5-6-7	180	25			
	6	C5	C1	6-7	0.47	1250						
	7	C6	C1									
BO	1	BOH	SP1	1-2	0.24	20000	1-2-3	93.375	25	5-2-4-1	120	100
	2	BC1	SC1	1-4	0.24	20000	1-2-5	93.375	25	5-4-2-3	120	300
	3	BC2	SC1	2-3	0.24	20000	1-4-3	93.375	25	3-4-2-1	120	100
	4	BC3	SC1	2-5	0.23	20000	1-4-5	93.375	25			
	5	BC4	SC1	3-4	0.21	20000	3-4-5	93.369	25			
				4-5	0.24	20000	3-2-5	93.369	25			
				2-4	0.3	constraints	4-5-2	65.684	25			
OST	1	OS1	SNa	4-5	0.43	1250	2-4-5	162.2	25	1-2-4-3	0	50
	2	OS2	SC4	3-6	0.43	1250	3-4-5	102.62	25	5-2-3-4	0.553	50
	3	OS3	SC4	6-7	0.43	5000	1-3-6	120.64	25	6-2-4-3	9.113	50
	4	OS4	SC4	1-2	0.27	constraints	2-3-6	162.9	25			
	5	OS5	SN0	1-3	0.27	constraints	4-3-6	116.48	25			
	6	OS6	SC3	2-3	0.27	constraints	3-6-7	115.86	45			
	7	OS7	C3	2-4	0.27	constraints						
				3-4	0.27	constraints						

2.2. Validation of the CG Models of Borneol and Osthole

In this study, the CG models of borneol and osthole are new models developed by our team. We calculated the bond distributions of molecules in water solution systems. The data was calculated in all atoms (AA) and coarse-grained (CG) levels to validate the accuracy of our CG models.

The AA simulations were performed on a GROMOS 54a7 by using a GROMACS software package (version 4.6.3). The SPC water model was also used to model the solvent. The LINCS algorithm was applied to constrain all bond lengths with a relative geometric tolerance of 10^{-4}. We conducted the CG simulation by the standard simulation protocol used in the Martini parametrization (available online: http://www.cgmartini.nl/).

The results shown in Figures S5 and S6 indicated that all the bond distributions of borneol and osthole in CG representations were similar to those in the AA representations, which demonstrated that our CG models are valid for further simulation studies.

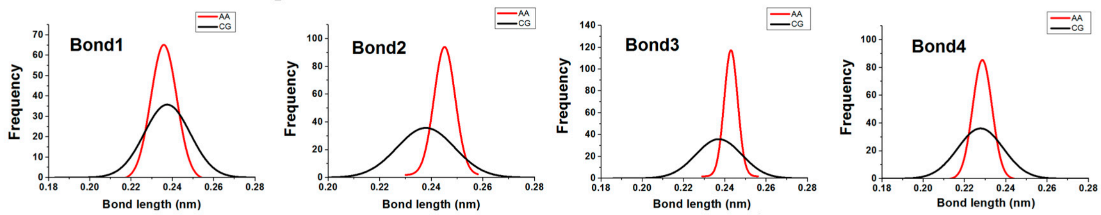

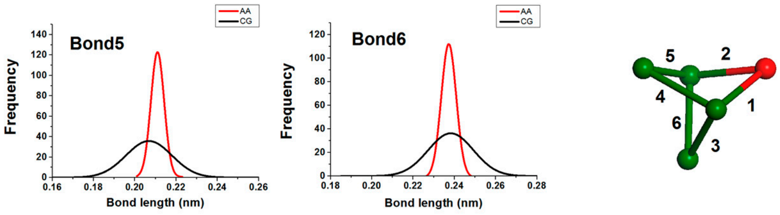

Table S2. Average bond length of BO from AA and CG simulations. (nm)

	AA	CG
Bond1	0.23580	0.23744
Bond2	0.24512	0.23784
Bond3	0.24291	0.23702
Bond4	0.22857	0.22769
Bond5	0.21099	0.20684
bond6	0.23727	0.23825

Figure S5. Bond distributions of borneol molecules calculated from AA and CG simulations.

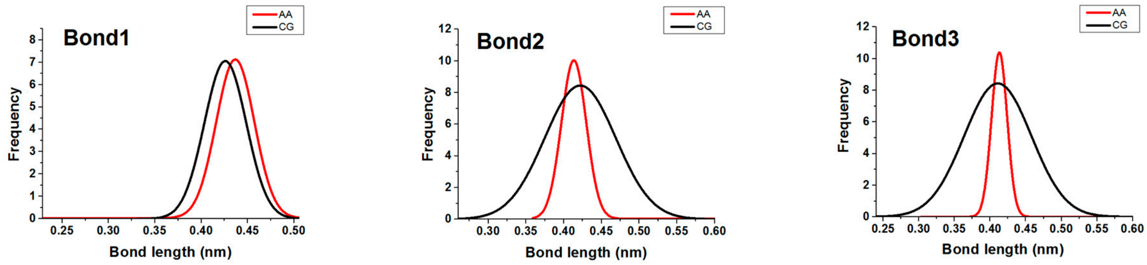

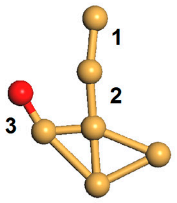

Table S3. Average bond length of OST from AA and CG simulations. (nm)

	AA	CG
Bond1	0.43661	0.42587
Bond2	0.41334	0.42185
bond3	0.41297	0.41075

Figure S6. Bond distributions of osthole molecules calculated from AA and CG simulations.

3. Structure Properties of Mixed-Bilayer System

The variation of the average area per lipid (APL) with temperature was calculated by an annealing simulation at a rate of 0.25 K/ns (Figure S7). The total simulation time was 600 ns. The phase transition temperature (Tm) of the mixed-bilayer system was in the range of 332.9–380.3 K. The

corresponding morphology changes are shown in Figure S8. Other structure properties are shown in Table S4.

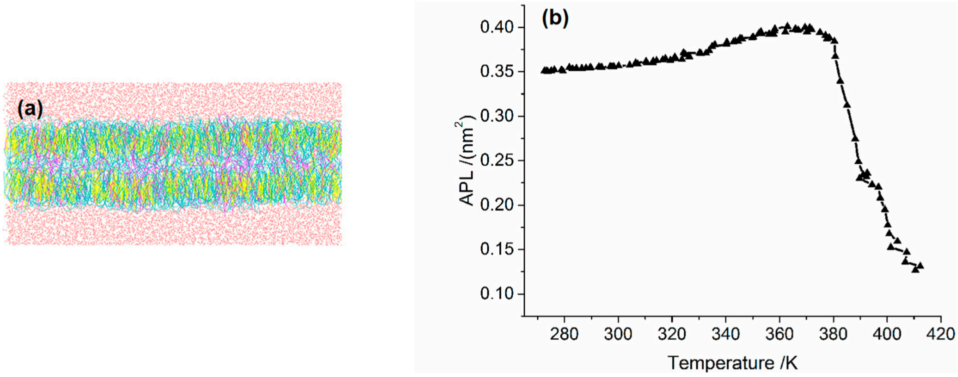

Figure S7. (**a**) Initial structure of mixed-bilayer system; (**b**) Variation of APL with temperature.

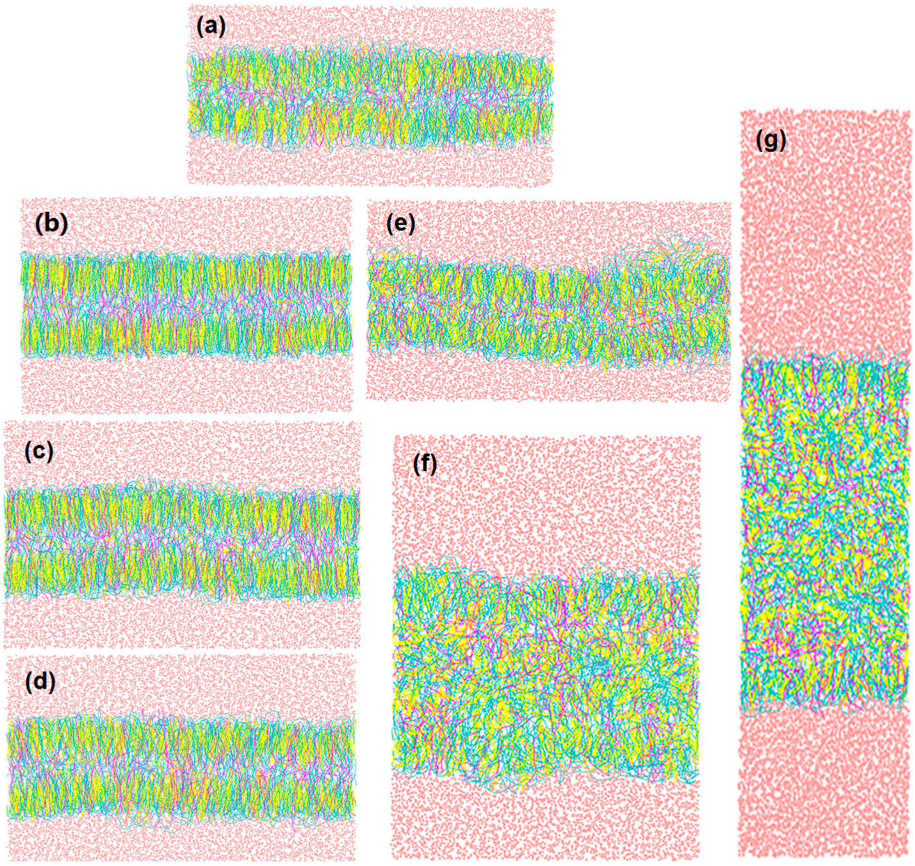

Figure S8. Morphologies of the mixed-bilayer system at (**a**) initial temperature; (**b**) 273 K; (**c**) 332.9 K; (**d**) 350.8 K; (**e**) 380.3 K; (**f**) 395.5 K; (**g**) 415 K.

Table S4. Structure properties of mixed-bilayer system.

**APL/nm^2**	**D_{H-H}**	**Tm/K**
0.3508	4.3144	332.9–380.3

4. Pre-MD Run of Bilayer System

The total energy of a typical system with 10% borneol and 10% osthole added to lipid bilayer is shown in Figure S9. The results indicated that 400 ns total simulation time was sufficient for the simulation systems to reach equilibrium.

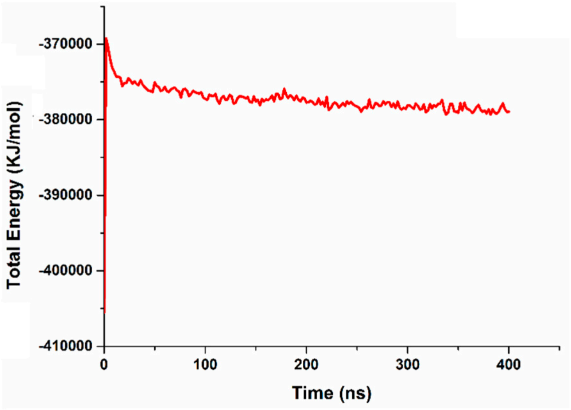

Figure S9. The total energy of a typical simulation system with 10% borneol and 10% osthole added to lipid bilayer during 400 ns at 310 K.
